# Bilateral Hypoglossal Nerve Palsy After Cardiac Surgery

**DOI:** 10.31486/toj.24.0126

**Published:** 2025

**Authors:** Amanda Vining, Jay E. Trusheim, Kelly G. Ural

**Affiliations:** ^1^ Department of Anesthesiology and Perioperative Medicine, Ochsner Clinic Foundation, New Orleans, LA; ^2^ The University of Queensland Medical School, Ochsner Clinical School, New Orleans, LA

**Keywords:** *Denervation*, *hypoglossal nerve*, *hypoglossal nerve injuries*, *postoperative complications*

## Abstract

**Background:**

Hypoglossal nerve palsy is a rare perioperative complication caused by excessive stretching of the nerve. Symptoms include tongue deviation, dysarthria, hoarseness, and dysphagia. We present the case of a patient who experienced bilateral hypoglossal nerve palsy after cardiac surgery.

**Case Report:**

A 68-year-old male with hypertension, sleep apnea, and aortic insufficiency presented for aortic valve replacement. He was easily intubated using video laryngoscopy, and surgery proceeded without incident. He remained intubated overnight and was extubated on postoperative day 1. Initially, hoarseness and tongue edema were noted. Further evaluation revealed oropharyngeal dysphagia, silent aspiration, and inability to protrude the tongue, all consistent with a bilateral hypoglossal nerve injury. Because the patient was unable to swallow, a percutaneous endoscopic gastrostomy (PEG) tube was placed. Three months later, electromyography showed denervation of the tongue, suggestive of hypoglossal nerve injury, with good prognosis for recovery. The PEG tube was removed, and the patient was able to tolerate a soft diet. Eight months postoperatively, the patient was started on a normal diet, and at 18 months postoperatively, his speech and vocal fatigue had improved to approximately 90% of normal.

**Conclusion:**

Although rare, hypoglossal nerve palsy is a perioperative complication that can have deleterious effects on patient well-being. Most cases are self-limited and resolve completely in 4 to 6 months; however, some patients experience more lasting effects. Anesthesiologists should take appropriate precautions when positioning a patient's head and neck during intubation and throughout the duration of surgery to help prevent hypoglossal nerve injury.

## INTRODUCTION

Hypoglossal nerve palsy is a rare perioperative complication with a reported incidence of 0.36% to 2.7% following direct or suspension laryngoscopy.^1^ Patients with bilateral hypoglossal nerve palsy present with tongue deviation, dysarthria, hoarseness, and dysphagia.^2-5^ Workup includes imaging to exclude stroke and a thorough examination to exclude endotracheal trauma or hematoma. There are no definitive risk factors for hypoglossal nerve palsy, but males and patients undergoing orthopedic or otolaryngologic operations are postulated to be at increased risk.^4^

We present a patient who developed bilateral hypoglossal nerve palsy after an aortic valve replacement with repeat sternotomy.

## CASE REPORT

A 68-year-old male (6′0″, 86.6 kg) with a history of hypertension, obstructive sleep apnea, previous mitral valve repair, and aortic valve insufficiency presented for aortic valve replacement with repeat sternotomy. Preoperatively, he mentioned a history of cervical spine pain from a motor vehicle accident 2 years prior. Workup at that time was concerning only for trapezius muscle strain; however, extra caution was taken on intubation to maintain the patient's neck in a neutral position despite his ability for full range of motion without pain during the preoperative examination.

After induction of anesthesia, a Cormack-Lehane grade 1 view was obtained using a McGRATH MAC video laryngoscope (Medtronic) with a size 3 blade. A 7.5-mm endotracheal tube (ETT) was placed; the cuff was inflated until an audible leak at 20 cm H_2_O was no longer detected and secured at 22 cm at the lips. A 9 French double lumen central line was placed in the right internal jugular vein without complication. A transesophageal echocardiogram (TEE) probe was inserted and left in place during the surgery for a total TEE time of approximately 4 hours. The surgery proceeded without incident or complication, and hemodynamics were adequately maintained throughout the procedure with minimal need for vasopressor agents. No blood products were administered. At the conclusion of the surgery when the TEE probe was removed, the anesthesiologist noted that the patient's tongue was slightly pale in color, but no other abnormalities or signs of trauma were noted.

The patient remained intubated overnight and was extubated on postoperative day 1. Upon extubation, significant tongue edema and hoarseness were noted. On postoperative day 2, a speech and language pathologist was consulted and determined that the patient had phonic difficulty in addition to tongue weakness and incomplete swallowing, thought to be attributable primarily to edema and suspected to resolve in the coming days. However, when little improvement was seen by postoperative day 5, Otolaryngology and Neurology were consulted.

Flexible laryngoscopy revealed “mildly decreased adduction of left true vocal fold” and the inability to protrude the tongue, which were concerning for bilateral hypoglossal nerve injury. Imaging, including magnetic resonance imaging of the brain and soft tissues of the neck, revealed mild edema of the vocal cords (Figure), but no other abnormalities and no infarcts were noted. The following day, modified barium swallow study demonstrated severe oropharyngeal dysphagia and silent aspiration of thin and thickened liquids, and the decision was made to place a percutaneous endoscopic gastrostomy (PEG) tube. The patient was discharged home on postoperative day 13.

**Figure.**
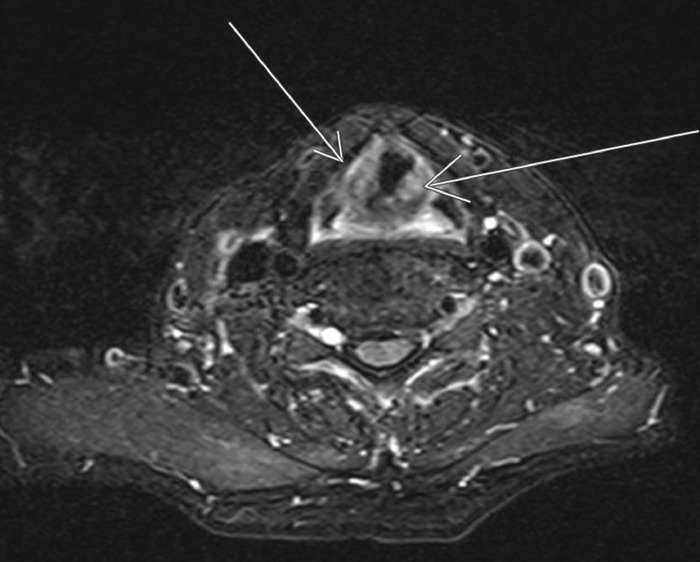
T2 noncontrast magnetic resonance imaging of soft tissue in the neck is positive for heterogeneous edema (arrows) of the vocal cords.

Two weeks postdischarge, the patient had mild recovery of tongue function. Electromyography 3 months later showed “active denervation … in the tongue bilaterally without significant change in motor unit morphology … [suggestive of] injury to the hypoglossal nerve with good prognosis for recovery.” Three months after his original surgery date, the patient underwent another flexible laryngoscopy that showed improvement in abduction and adduction of the left vocal cord in addition to fasciculations of the left side of the palate. Three and a half months after the PEG tube was placed, the patient's swallowing improved, he was able to tolerate a soft diet, and the PEG tube was removed. Eight months postoperatively, the patient was able to tolerate a normal diet; however, he continued speech and voice therapy for hoarseness and vocal fatigue. At his most recent follow-up, approximately 18 months postoperatively, the patient reported that his voice was approximately 90% recovered and he was continuing to work with speech therapy.

## DISCUSSION

Isolated hypoglossal nerve injuries have been reported in multiple case studies involving shoulder arthroscopies, tonsillectomies, cardiac surgeries, and other procedures.^2-5^ Multiple mechanisms have been described as potential causes, but the common thread is a stretch-type injury to the nerve.^4^ The hypoglossal nerve exits the skull at the hypoglossal canal near the foramen magnum, descends caudally along the internal and external carotid artery and the internal jugular to the angle of the mandible where it travels anteriorly to the greater cornu of the hyoid bone to the submandibular region along the inferior aspect of the tongue.^4,6^ During head and neck positioning for laryngoscopy, distal fibers supplying the tongue can be sheared, in addition to the potential for hypoglossal nerve stretching as the nerve exits the vertebral column.

Neither fiber shearing nor nerve stretching is likely to have occurred during intubation of our patient because the endotracheal tube was easily placed with video laryngoscopy while the head was maintained in a neutral position; however, prior to the start of surgery, a shoulder roll was placed to improve surgical exposure. Use of a shoulder roll can extend the neck, causing a position change from the neutral midline that could compress the hypoglossal nerve against the C1 vertebra or ETT.^2^ In addition, males have a higher chance of hypoglossal nerve injury during procedures.^4^ A proposed mechanism for this increased risk is the longer length of the greater cornu and larger hyoid bone found in males vs females. Because of the hypoglossal nerve pathway, the nerve may be vulnerable to outside forces such as anterocaudal movement.^4,6-8^ Compression of the hypoglossal nerve by the TEE probe is another possible etiology as the TEE probe could theoretically increase the force of the ETT cuff onto the nerve by displacement.^2,5^ Overinflation of the ETT cuff can also cause compression of both the recurrent laryngeal and hypoglossal nerves, especially when the cuff is positioned higher in the airway.^2^

Most cases of hypoglossal nerve palsy are diagnosed in the acute postoperative period. Prolonged intubation can lead to a delay in the discovery of symptoms and could be another risk factor for injury. Initial evaluation of patients should include a multidisciplinary workup to differentiate central vs peripheral causes.^5^

Most patients with mild symptoms can expect a symptom-free recovery in approximately 4 to 6 months^5^; however, our patient continued to have symptoms for much longer, and at 18 months postoperatively, he was not completely symptom-free. Minimal research has been done in recovery methods for peripheral nerve injuries such as hypoglossal nerve injury. Studies of steroid use and vocal cord injections are inconclusive at best. Although some clinical data support the idea that steroid administration may reduce nerve edema following injury, a study by Lachanas et al showed no benefit from steroids after postthyroidectomy voice changes.^9^ Another study suggested that steroids could inhibit recovery by inhibiting toll-like receptors necessary for Wallerian degeneration and phagocytosis of myelin debris.^10^

Measures to help prevent peripheral nerve injuries include minimally occlusive cuff inflation, proper tube fixation to decrease excessive tube motion, gentle intubation with minimal external force, careful head and neck maneuvers, and neutral positioning of the head and neck.^4^ The approach to treatment should be multidisciplinary, with a focus on speech, language, and swallowing.^11^

## CONCLUSION

Hypoglossal nerve palsy is a rare perioperative complication that can have deleterious effects on patient well-being. A multidisciplinary treatment approach is necessary. Anesthesiologists should be aware of this potential complication and use caution when positioning a patient's head and neck during intubation and throughout the duration of lengthy surgeries.

## Notes

*This article meets the Accreditation Council for Graduate Medical Education and the American Board of Medical Specialties Maintenance of Certification competencies for Patient Care and Medical Knowledge.*
